# Mental Health and Substance Use of Farmers in Canada during COVID-19

**DOI:** 10.3390/ijerph192013566

**Published:** 2022-10-19

**Authors:** Rochelle Thompson, Briana N. M. Hagen, Margaret N. Lumley, Charlotte B. Winder, Basem Gohar, Andria Jones-Bitton

**Affiliations:** 1 Department of Population Medicine, University of Guelph, Guelph, ON N1G2W1, Canada; 2 Department of Psychology, University of Guelph, Guelph, ON N1G2W1, Canada

**Keywords:** agriculture, anxiety, depression, burnout, resilience, perceived stress, one health

## Abstract

Farmers in Canada faced higher levels of mental distress than the general public prior to the Coronavirus Disease 2019 (COVID-19) pandemic and are generally less likely than the public to seek help. However, the mental health impacts of COVID-19 on farmers in Canada remain unexplored. Our objective was to investigate mental health outcomes among farmers in Canada by gender and within the context of COVID-19. We conducted a national, online, cross-sectional survey of farmers in Canada (February–May 2021). The survey included validated scales of anxiety, depression, perceived stress, burnout (emotional exhaustion, cynicism, professional efficacy), alcohol use, resilience, and questions regarding participants’ perceived changes in these outcomes during the pandemic. Data were also collected on the impact of COVID-19 specific social and economic factors on mental health, help-seeking, and sense of community belonging through the pandemic. Descriptive statistics were summarized, and Chi-square analyses and t-tests were conducted to compare survey results between genders and to data collected in our similar 2016 survey and normative population data. A total of 1167 farmers participated in the survey. Participants scored more severely across scales than scale norms and the general Canadian population during COVID-19. Scale means were consistent between the 2016 and 2021 samples. Most participants with moderate to severe scores for any outcome reported worsening symptoms since the pandemic began. Women fared significantly worse than men across measures. Over twice as many women reported seeking mental health or substance use support during the pandemic than men. Participants rated the mental health impacts of all social and economic factors related to COVID-19 examined significantly (*p* < 0.05) differently than the Canadian public. The pandemic has negatively impacted the mental health of farmers in Canada and in ways that differ from the general population. National level and gender-specific mental health supports are needed to help improve the mental health of farmers in Canada.

## 1. Introduction

Ongoing assessment of the mental health impacts of the Coronavirus Disease 2019 (COVID-19) pandemic is important, as the impact of factors affecting the public (i.e., public health restrictions, grocery shortages due to panic buying, personal protective equipment shortages, school closures, fear of contracting COVID-19, and uncertainty) may be cumulative [1]. Indeed, Lowe et al. (2022) found the mental health of Ontarians and Albertans deteriorated over the beginning of the pandemic (Spring 2020), improved through Summer 2020, and declined again in the Fall/Winter of 2020/2021 [2]. Mental Health Research Canada’s national polling found levels of anxiety and depression were higher in late 2020/early 2021 than during the first wave of the pandemic in Spring 2020 [3].

In addition to the COVID-19 factors impacting Canadians in the Winter/Spring of 2021, such as continuing to experience public health restrictions, school closures, mask mandates, and the start of vaccine rollout, Canadian farmers faced unique COVID-19 impacts. In early 2021, workplace outbreaks of COVID-19 in meat processing facilities left some farmers with nowhere to send their livestock [4]. Fruit and vegetable farmers relying on essential temporary international agricultural workers were concerned about coordinating travel restrictions, COVID testing, and housing requirements for the 2021 season after facing labour shortages in 2020 [5,6,7]. Uncertainty surrounding the global pandemic added to stress when forecasting markets [8], as COVID-19 outbreaks around the world impacted the global economy [9]. These pandemic-related stressors were in addition to farming stressors at the time, such as potential for African Swine Fever [10] and forecasted weather concerns (e.g., drought in prairies) [11,12]. The impacts of these pandemic-associated stressors on the mental health of farmers in Canada are unknown; however, there is evidence that the mental health of farmers around the world has been negatively impacted by the pandemic. For example, in a study of mental health in rural residents of China during COVID-19 lockdowns, Jia et al. [13] found rural women and those whose primary income came from agricultural production had higher risk of mental illness.

The first national survey of farmer mental health in Canada was conducted in 2015/2016 and found that prior to the COVID-19 pandemic, farmers in Canada faced increased levels of stress, anxiety, depression, burnout, and lower resilience and help-seeking than population norms [14,15]. Since the 2016 survey, mental health awareness campaigns and mental health literacy programs in the agricultural community have been launched [16]. For example, “In the Know”, a mental health literacy training program tailored to Canadian agriculture and delivered by mental health professionals with agricultural backgrounds, has been introduced in 5 provinces (Ontario, Alberta, Nova Scotia, British Columbia, Manitoba) and delivered to approximately 900 members of the Canadian agricultural community thus far [17,18]. Given the increase in mental health awareness programs since 2016 and the start of the COVID-19 pandemic, we anticipate the mental health landscape of farmers in Canada has changed since 2016. The primary goal of the present study was to determine the prevalence of anxiety, depression, stress, burnout, alcohol use, and resilience among farmers in Canada in the context of COVID-19, and the perceived impact of the pandemic on the mental health of farmers in Canada.

A secondary goal of this study was to explore gender differences in farmers’ mental health during COVID-19. Results from international studies have found that the mental health impacts of the pandemic are gendered [19]; compared to men, women have scored more severely on mental health measures in China [20], Italy [21], United Kingdom [22], and Canada [23] during COVID-19. Globally, more women than men are affected by deleterious domestic violence and financial disadvantage, which have been exacerbated during the pandemic [24]. Women have also been affected by particular social and economic aspects of life during the pandemic such as childcare and household duties, which increased during the pandemic due to school closures and stay-at-home orders [25]. Farming women have historically taken on a disproportionate amount of unpaid labour, such as childcare and household maintenance [26], in addition to on-farm and potentially off-farm labour. As school closures during COVID-19 in Canada meant higher caregiving responsibilities, farming women may have had a greater inter-role conflict (e.g., between farmer and caregiver) than men [27]. Therefore, we aimed to investigate whether Canadian farming women faced more negative mental health impacts of the pandemic than farming men.

Finally, there has been some investigation of factors which mitigate negative mental health impacts during the pandemic and associated lockdowns. Notably, community characteristics (e.g., available services, cohesion, green space) have been documented in many countries [13,28,29] to affect mental health during pandemic lockdowns, particularly in rural communities [13,29]. We sought to learn how pandemic related factors were perceived by farmers to impact their mental health, and specifically whether the pandemic impacted farmers’ sense of community belonging.

## 2. Materials and Methods

### 2.1. Study Design

We conducted a cross-sectional study from 1 February to 31 May 2021. Farmers were invited to complete an online questionnaire via Qualtrics (Provo, UT, USA). We used convenience sampling to recruit participants through use of national and provincial agricultural organizations who promoted the questionnaire in their newsletters, social media, and member listervs. The researchers also posted the questionnaire on social media using hashtags (e.g., #CdnAg, #AgTwitter).

To participate, participants were required to be over 18 years of age, able to read and write English or French, and self-identify as a farmer in any commodity in Canada. As an incentive, participants could enter their email address on a separate Qualtrics survey to enter a draw to win one of five $200 prizes. Email addresses were not connected to responses to the main survey, and no identifying information was asked in the study questionnaire. Informed written consent was obtained before participants began the questionnaire. This study protocol was approved by the Research Ethics Board at the University of Guelph (21-01-001).

### 2.2. Questionnaire

We used validated scales to investigate six outcomes: anxiety, depression, perceived stress, burnout (emotional exhaustion, cynicism, & professional efficacy), alcohol use, and resilience. Following each measure, participants were asked a question adapted from Statistics Canada’s Canadian Survey on COVID-19 and Mental Health, “Taking them together, did the above statements relating to [outcome] apply to you more over the past month than before the pandemic, less than before the pandemic, or about the same as before the pandemic?” with a 7-point Likert-type response option (1 = a lot more; 7= a lot less). To measure the mental health impacts of COVID-19 related variables (e.g., social isolation, daily COVID-19 news), we used questions from Mental Health Research Canada’s Mental Health During COVID-19 Outbreak polls and Statistics Canada’s Canadian Survey on COVID-19 and Mental Health. The questionnaire also collected data on demographics and farming taken from the 2016 cycle version. In all, the questionnaire was estimated to take 20–25 min to complete.

#### 2.2.1. Anxiety and Depression

The Generalized Anxiety Disorder-7 (GAD-7) and Patient Health Questionaire-9 (PHQ-9) were used to measure anxiety symptom severity [30] and depressive symptom severity [31], respectively. The seven items on the GAD-7 and nine on the PHQ-9 are based on the DSM-IV diagnostic criteria for anxiety disorders and depressive disorders, respectively [31,32]. Neither scale has demonstrated differential item functioning across language (including English and French), age, or sex of participants [33]. These scales have established validity and reliability as measures of depression and anxiety [34]. Additionally, the PHQ-9 has been endorsed by the National Institute for Health and Clinical Excellence “for use in primary care in measuring baseline depression severity and responsiveness to treatment” [31]. Both scales require participants to self-report their anxiety or depressive symptoms over the past two weeks on a 4-point Likert-type scale, from 0 (not at all) to 3 (nearly every day) [35,36]. The GAD-7 is scored within a range of 0–21, where scores indicate how likely the participant is to have no (0–5), mild (6–10), moderate (11–15), or severe (16–21) anxiety disorder symptoms [32]. The PHQ-9 is scored within a range of 0–27, where scores indicate how likely the participant is to have no (0–4), mild (5–9), moderate (10–14), moderate to severe (15–19) or severe (20–27) depressive disorder symptoms [37]. Using cut points of >10, the GAD-7 and PHQ-9 scales are used to screen for Generalized Anxiety Disorder (GAD) and Major Depressive Disorder (MDD), respectively [33].

#### 2.2.2. Perceived Stress

The Perceived Stress Scale (PSS) 10-item version was used to measure perceived stress in this sample. The PSS was developed with the aim to measure “the degree to which situations in one’s life are appraised as stressful” [38]. It has established reliability and validity in English [39] and French [40], and has been previously used with Canadian farmers [15]. The 10-item scale asks participants to rate their thoughts and feelings relating to perceived stress within the last month on a 5-point Likert type scale from 0 (never) to 4 (very often) [38]. Higher mean scores indicate higher perceived stress [38].

#### 2.2.3. Burnout

We used the Maslach Burnout Inventory-General Survey (MBI) to measure the three components of burnout: emotional exhaustion, “wearing out, loss of energy, depletion, debilitation, and fatigue”; cynicism, “negative or inappropriate attitudes towards clients, irritability, loss of idealism, and withdrawal”; and professional efficacy, “productivity or capability, morale, and an ability to cope” [41]. The MBI is considered the gold standard to measure the construct of burnout and its components [42]. The scale has established validity and reliability in English [41] and French [43] and has been used previously with Canadian farmers [14]. The 16-item scale asks participants to rate their thoughts and feelings relating to burnout on a 7-point Likert-type scale from 0 (never) to 6 (every day) [41]. Responses for each of the three subscales (emotional exhaustion, cynicism, and professional efficacy) were scored separately. Higher mean scores for each subscale indicate higher emotional exhaustion, cynicism, or professional efficacy.

#### 2.2.4. Alcohol Use

We used the Alcohol Use Disorders Identification Test (AUDIT) to screen for unhealthy alcohol use. The AUDIT was developed by the World Health Organization (WHO) to measure “alcohol consumption, drinking behaviour, and alcohol-related problems” [44]. It has been validated in English [45] and French [46]. The 10-item scale is scored from 0 to 40, where a score of 0 indicates the respondent has never drank alcohol, 1–7 indicates low risk consumption, 8–14 indicates hazardous/harmful alcohol consumption, and 15–40 indicates likely moderate-severe alcohol use disorder (AUD) [44,47].

#### 2.2.5. Resilience

The Connor Davidson Resilience Scale (CD-RISC) 10-item version was used to investigate resilience. The CD-RISC was developed to measure resilience as “persistence and hardiness” [48]. Though there is no gold standard measure of resilience [49], the CD-RISC is one of the most widely used resilience measures [50] and has been validated in the general population in both English and French [51]. The CD-RISC 25-item version has previously been used with farmers in Canada [15], but the 10-item version has since been recommended for use in research due to superior psychometrics [50]. The measure asks participants to rate their agreement with 10 statements in the past month on a 5-point Likert-type scale from 0 (not true at all) to 4 (true nearly all the time). Higher mean scores indicate greater resilience [51].

#### 2.2.6. Mental Health Impacts of COVID-19 Factors

To measure the perceived impact of various COVID-19-specific factors on participants’ mental health (e.g., social isolation, difficulties getting groceries), we used 15 items from Mental Health Research Canada’s Mental Health During COVID-19 Outbreak polls [3,52] (Table 1). Participants were asked to rate the impact of the 15 factors on their mental health using a 5-point Likert-type scale from 1 (very negative) to 5 (very positive). Each item was individually reported.

#### 2.2.7. Self-Rated Mental Health & Perceived Stability/Change in Mental Health

To assess general self-rated mental health, we used one item from Statistics Canada’s Canadian Survey on COVID-19 and Mental Health. Participants were asked, “In general, how is your mental health?” with response options “Excellent”, “Very good”, “Good”, “Fair”, and “Poor” [53]. Participants who answered either “Excellent” or “Very Good” options were said to have high self-rated mental health [54]. To measure perceived stability/change in mental health, participants answered one item from Statistics Canada’s *Canadian Survey on COVID-19 and Mental Health;* “Compared to before the COVID-19 pandemic, how would you say your mental health is now?” with response options: “Much better now”, “Somewhat better now”, “About the same”, “Somewhat worse now” and “Much worse now” [53]. Participants who answered “Much better now”, “Somewhat better now”, or “About the same” were said to have stable/improved mental health [54].

#### 2.2.8. Community Belonging & Perceived Stability/Change in Community Belonging

To investigate community belonging, we used one item from Statistics Canada’s *Canadian Survey on COVID-19 and Mental Health.* Participants were asked, “How would you describe your sense of belonging to your local community?” with response options “Very strong”, “Somewhat strong”, “Somewhat weak”, and “Very weak” [53]. Participants with high community belonging included those who answered “Very strong” or “Somewhat strong” [54]. To measure perceived stability/change in community belonging, participants answered one item developed by the researchers to match the item measuring stable/improved mental health from Statistics Canada’s *Canadian Survey on COVID-19 and Mental Health*; “Compared to before the COVID-19 pandemic, how would you say your sense of belonging to your local community is now?” with response options: “Much better now”, “Somewhat better now”, “About the same”, “Somewhat worse now” and “Much worse now”.

#### 2.2.9. Help Seeking during COVID-19

To investigate help seeking during COVID-19, participants answered one item from Statistics Canada’s *Canadian Survey on COVID-19 and Mental Health*; “Have you accessed any resources (on the internet, via phone, or in-person) to help you manage your emotions, mental health, or use of alcohol or drugs during the COVID-19 pandemic?” with response options of “Yes” or “No” [53].

### 2.3. Sample Size

A minimum required sample size of 385 was calculated using a conservative a priori prevalence estimate of 50%, an allowable error of 5%, and a confidence level of 95%.

### 2.4. Statistical Analyses

We used descriptive statistics (means, standard deviations (SD), percentages, tertiles) of participant demographics to examine the data. To increase statistical power, person-mean imputation was used to account for missing data in validated scales (GAD-7, PHQ-9, CD-RISC, PSS, MBI, AUDIT) if only one response was missing [55]; if a participant was missing two or more responses to a scale/subscale, they were omitted from analysis of that scale/subscale. For the validated scales used (GAD-7, PHQ-9, CD-RISC, PSS, MBI, AUDIT), participants’ total scores were calculated, and scores were categorized according to each scale’s manual. As the data were normally distributed, Chi-square tests were used to compare proportions and t-tests were used to compare means between genders and to test comparisons of the 2021 survey data to population norms and 2016 survey data, where available. Results were also compared to Mental Health Research Canada’s *Mental Health During COVID-19 Outbreak* poll 6 (collected April 20 to 28, 2021), and Statistics Canada’s *Canadian Survey on COVID-19 and Mental Health* cycle 2 (collected February to May 2021), where noted. Statistical significance was set to *p* < 0.05. All statistical analyses were conducted using R version 4.1.1.

## 3. Results

### 3.1. Study Population

A total of 1167 farmers participated in the study. As participants were not required to answer any questions beyond consent, the sample size differed throughout the survey as noted (range *n* = 948–1167). The average age of participants (*n* = 968) was 49.3 years (SD 13.7; IQR 38–60; range 20–93). The majority (70.6%, *n* = 824) of participants chose to complete the survey in English, and 60.7% of participants were men (*n* = 565). Additional details of the sample population are presented in Table 2.

### 3.2. Prevalence of Mental Health Outcomes

Mean scale scores, comparisons by gender, and comparisons to each scale’s normative population data are presented in Table 3.

#### 3.2.1. Anxiety

**Figure 1:**
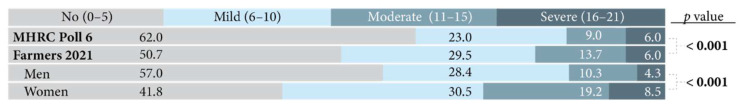
Classification of no, mild, moderate, and severe anxiety disorder symptoms for overall 2021 sample and by gender, in comparison to the Canadian general population during COVID-19 from Mental Health Research Canada’s (MHRC) Mental Health During COVID-19 Outbreak poll 6.

The average GAD-7 score for 2021 farmers was 6.12 (SD 4.88) and was significantly higher than the GAD-7 normative population mean (2.97 (3.35); *p* < 0.001) [32]. A positive screen for GAD, indicated by a score >10 on the GAD-7, was significantly higher in 2021 farmers (26.8%) compared to scores reported for Canadians in Spring 2021 (15.0%, *p* < 0.001) and Fall 2020 (13.0%, *p* < 0.001) (Table 4) [57]. Among the 2021 study population, 6% reported scores indicative of symptoms of severe anxiety disorder, consistent with Canadians in Spring 2021 (6%) (Figure 1) [3]. However, significantly more 2021 farmers demonstrated symptoms of moderate and mild anxiety disorder (13.7% and 29.5%, respectively) compared to Canadians in Spring 2021 (9.0% and 23.0%, respectively) [3]. Out of the 2021 participants, women had higher mean GAD-7 scores than men (7.16 vs. 5.45, respectively, *p* < 0.001), more positive screens for GAD (34.9% vs. 21.0%, *p* < 0.001), and more women demonstrated symptoms of mild (30.5%), moderate (19.2%), and severe GAD (8.5%) than men (28.4%, 10.3%, and 4.3% respectively; *p* < 0.001).

#### 3.2.2. Depression

**Figure 2:**
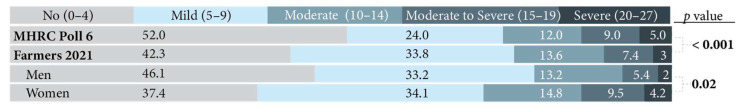
Classification of no, mild, moderate, moderate to severe, and severe depressive disorder symptoms for overall 2021 sample and by gender, in comparison to the Canadian general population during COVID-19 from Mental Health Research Canada’s (MHRC) Mental Health During COVID-19 Outbreak poll 6.

The mean PHQ-9 score for 2021 farmers was 5.89 (SD 5.27), which was significantly higher than the PHQ-9 general population scale norm (2.91, *p* < 0.001) [37]. The prevalence of a positive MDD screen (i.e., PHQ-9 score >10) was significantly higher in 2021 farmers (23.6%) than Canadians in the Spring of 2021 (19.0%, *p* < 0.001), Canadians in the Fall of 2020 (15.2%, *p* < 0.001), and Canadians prior to the pandemic (6.7%, *p* < 0.001) (Table 5) [58]. Fewer 2021 farmers demonstrated symptoms of severe (2.8% vs. 5.0%) and moderate to severe depressive disorder (7.4% vs. 9.0%) than Canadians in Spring 2021, but more 2021 farmers demonstrated symptoms of moderate (13.6% vs. 12.0%) and mild depressive disorder (33.8% vs. 24.0%) than Canadians in Spring 2021 (Figure 2) [3]. The mean PHQ-9 score in 2021 farmers was significantly higher for women (6.87) than for men (5.25, *p* < 0.001). Of the 2021 participants, women also had a higher prevalence of positive MDD screens than men (28.1% vs. 20.5%), and more women demonstrated symptoms of mild, moderate, moderate to severe, and severe depressive disorder than men (*p* = 0.02).

#### 3.2.3. Perceived Stress

The average PSS score of the 2021 farmers was 18.72 (SD 7.03), which is significantly higher than the normative population mean (13.0, (SD 6.34); *p* < 0.001) [59], but not significantly different from the 2016 sample mean (18.9 (4.9); *p* = 0.48) [15]. Of the 2021 participants, the mean PSS score for women (20.46, SD 6.98) was significantly higher than that for men (17.46, SD 7.15). The mean PSS scores for man and woman genders in the 2021 sample (17.46 and 20.46) were significantly higher than the normative population means (13.7, SD 6.60 and 12.1, SD 5.90; *p* < 0.001) [59]. The mean PSS scores of women were not significantly different between the 2021 and 2016 farming samples (*p* = 0.44), but the mean PSS scores of men were significantly lower in the 2021 sample (17.46, SD 7.15) compared to the 2016 sample (18.3, SD 4.9; *p* = 0.02), although this difference is small (0.84 points) [15].

#### 3.2.4. Burnout

The average emotional exhaustion, cynicism, and professional efficacy subscale scores of the 2021 participants were 2.64 (1.62), 2.20 (1.45), and 4.70 (1.11), respectively. The emotional exhaustion and cynicism subscale means were significantly higher in the 2021 participants than normative population means (2.26 (1.47) and 1.74 (1.36); *p* < 0.001) [41]. The professional efficacy subscale mean was significantly higher in the 2021 sample than the normative population mean (4.34 (1.17); *p* < 0.001) [41], but significantly lower than the 2016 sample mean (4.85 (1.01); *p* = 0.001) [14]. Emotional exhaustion and cynicism subscale means did not significantly differ between the 2021 and 2016 farming samples. Within the 2021 participants, the mean emotional exhaustion subscale score of women (2.92, SD 1.62) was significantly higher than that of men (2.46, SD 1.60; *p* = 0.02), although this difference was small (0.9 points).

#### 3.2.5. Alcohol Use

**Figure 3:**
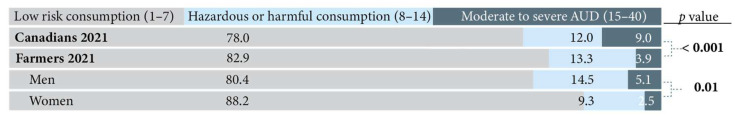
Classification of low risk consumption, hazardous or harmful consumption, and moderate to severe alcohol use disorder (AUD) for the overall 2021 sample and by gender, in comparison to the Canadian general population during COVID-19 from the Canadian Centre on Substance Use and Addiction and Mental Health Commission of Canada’s Mental Health and Substance Use During COVID-19 survey.

Participants in 2021 had significantly higher mean AUDIT scores (4.46) than the general population (3.61, *p* < 0.001) [60], reflecting higher alcohol consumption and related problems. Fewer farmers in 2021 were classified as having moderate to severe Alcohol Use Disorder (AUD) (3.9%) than Canadians in Fall 2020 (9.0%) [61], but more farmers in 2021 were classified as exhibiting hazardous or harmful alcohol consumption (13.3%) than Canadians (12.0%) in Fall 2020 (Figure 3) [61]. Within the 2021 sample, men had higher mean AUDIT scores than women (4.86 vs. 3.73, *p* < 0.001), and significantly more farming men were classified as having hazardous or harmful consumption or moderate to severe alcohol use disorder than farming women (*p* = 0.01). However, the 2021 farming men’s mean AUDIT scores did not significantly differ from the general population (*p* = 0.06), whereas the 2021 farming women’s mean AUDIT scores were significantly higher than women in the general population (3.73 vs. 2.71, *p* < 0.001) [60].

#### 3.2.6. Resilience

The mean CD-RISC score of the 2021 sample was 24.7 (SD 6.2), which was significantly lower than the mean CD-RISC score in the general population (31.8, SD 5.4, *p* < 0.001) [62]. Within the 2021 sample, the mean CD-RISC score of women (24.1) was significantly lower than that of men (25.0, *p* = 0.02), although this difference was small (0.9 points). Approximately 83.4% of participants in 2021 had CD-RISC scores lower than the reference population norm (<31.8); 92.5% of men and 86.5% of women in the 2021 sample had lower mean CD-RISC scores than the normative population (means of 33.53 and 31.08 versus 25.04 and 24.10, respectively) [62].

### 3.3. Perceived COVID-19 Impacts on Mental Health Outcomes

#### 3.3.1. Anxiety

**Figure 4:**
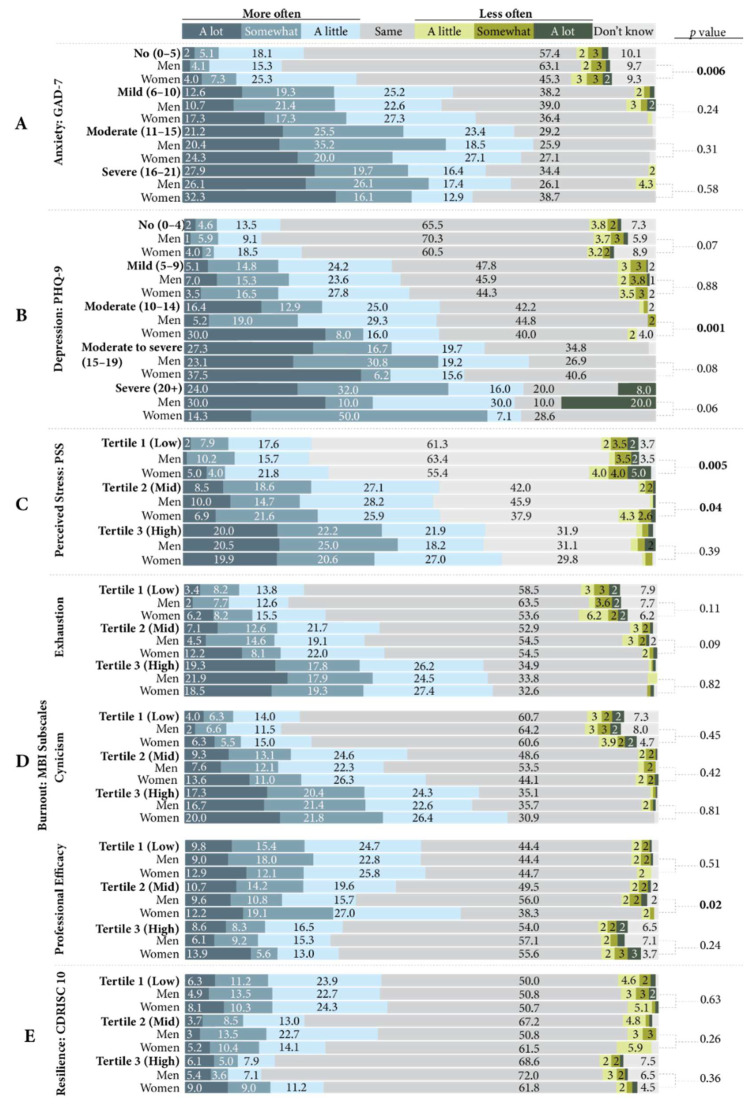
Perceived change in scale symptoms since the pandemic began by scale classification (Generalized Anxiety Disorder-7 (GAD-7) and Patient Health Questionnaire-9 (PHQ-9) or tertile-Perceived Stress Scale (PSS), Maslach Burnout Inventory–General Survey (MBI), Connor Davidson Resilience Scale-10 (CD-RISC 10)), and comparisons by gender.

Over half of participants who screened positive for GAD reported experiencing symptoms of anxiety more often since the pandemic began (Figure 4, Block A). Of those who were categorized as likely to have a severe anxiety disorder, 27.9% reported experiencing anxiety symptoms “a lot” more often since the pandemic began. More women than men who were classified as having no likely anxiety disorder reported increased anxiety symptoms since the pandemic began (*p* = 0.006).

#### 3.3.2. Depression

Approximately 54.3% of participants who demonstrated symptoms of moderate depressive disorder, and 63.7% and 72% of participants who demonstrated symptoms of moderate to severe and severe depressive disorder, respectively, reported experiencing more depression symptoms since the pandemic began (Figure 4, Block B). Women who demonstrated symptoms of moderate depressive disorder reported experiencing depression symptoms since the pandemic began significantly more often than men (*p* = 0.001).

#### 3.3.3. Perceived Stress

Most of the participants who had PSS scores in the mid- and high-tertiles reported feeling increased perceived stress since the pandemic began (54.2% and 64.1%, respectively) (Figure 4, Block C). More women in the low- and mid-tertiles reported feeling perceived stress less frequently since the pandemic began than men (*p* = 0.005 and *p* = 0.04, respectively).

#### 3.3.4. Burnout

In all three MBI subscales, most participants reported no change in their symptoms of burnout since the pandemic began (Figure 4, Block D). However, most participants with high emotional exhaustion (63.3%), cynicism (62.0%), and low professional efficacy (49.9%) reported experiencing symptoms of burnout more often since the pandemic began. Women with mid professional efficacy scores reported experiencing symptoms of burnout more often since the pandemic began significantly more than men (58.2% vs. 36.1%, *p* = 0.02).

#### 3.3.5. Resilience

Most of the participants in all three tertiles of CD-RISC scores reported no change in their levels of resilience since the pandemic began, and no significant gender differences were found (Figure 4, Block E).

#### 3.3.6. Alcohol Use

**Figure 5:**
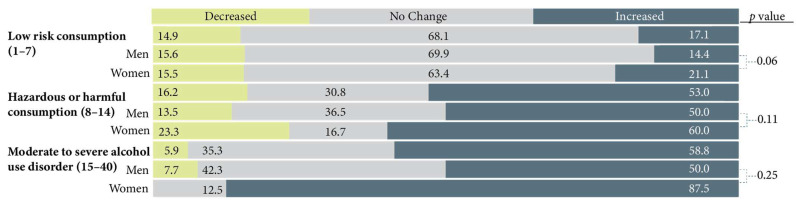
Perceived change in alcohol consumption since the pandemic began by Alcohol Use Disorder Identification Test (AUDIT) classification and by gender.

Perceived change in alcohol consumption since the pandemic began by AUDIT score category are presented in Figure 5. Approximately 3 out of 5 farmers (58.8%) who were classified as having moderate to severe AUD and 1 in 2 farmers (53%) who were classified as exhibiting hazardous or harmful alcohol consumption reported increased alcohol consumption since the pandemic began. No significant gender differences were found regarding the change in alcohol consumption since the pandemic began.

### 3.4. Impact of COVID-19 Specific Factors on Mental Health

**Figure 6:**
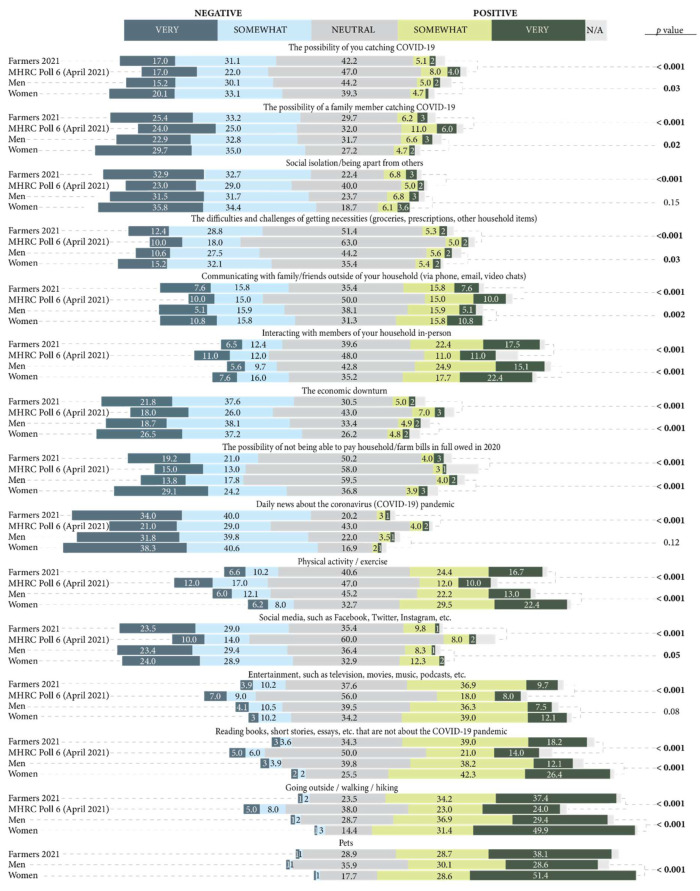
Perceived impact of COVID-19 specific factors on participants’ mental health in comparison to the general Canadian population during COVID-19 and comparison of the 2021 sample by gender (men and women lines).

Figure 6 shows the perceived impact of COVID-19 specific factors on participants’ mental health, with comparisons to the Canadian general population in Spring 2021 [3]. The COVID-19 specific factors which most farmers reported to have had a negative impact on their mental health included the possibility of a family member contracting COVID-19, the economic downturn, social media, and social isolation. Social isolation was the leading stressor on the mental health of Canadians, whereas daily news about the COVID-19 pandemic was the leading stressor on the mental health of farmers, where 74% of participants reported a negative impact. Conversely, over 50% of farmers reported many factors as having a positive impact on their mental health, including reading materials that are not related to COVID-19 and pets. Going outside had the most positive impact on both farmers and Canadians’ mental health in Spring 2021 [3].

More women than men reported difficulties with getting necessities (such as groceries) during the pandemic (*p* = 0.04) and the possibility of not being able to pay household/farm bills in 2020 (*p* < 0.001) as having a negative impact on their mental health during the pandemic. Further, more women than men reported physical activity (*p* < 0.001), reading material not about the COVID-19 pandemic (*p* < 0.001), going outside (*p* < 0.001), and pets (*p* < 0.001) as having a positive impact on their mental health during the pandemic.

### 3.5. Mental Health and COVID-19

Prevalence of high self-rated mental health and perceived stability/improvement of mental health since the pandemic began are presented in Table 6. Overall, 46.3% of farmers reported high self-rated mental health, which is significantly lower than Canadians in Spring 2021 (51.5%, *p* = 0.001) and Fall 2020 (59.9%, *p* < 0.001) [54]. Significantly more men (52.8%) reported high self-rated mental health than women (36.4%, *p* < 0.001), as well as stable/improved mental health since the pandemic began than women (69.4% vs. 60.3%, *p* < 0.001). One-third (66%) of farmers reported stable or improved mental health since the pandemic began, which is significantly higher than Canadians in Spring 2021 (58.1%, *p* < 0.001), but not significantly different from Canadians in Fall 2020 (66.5%, *p* = 0.72) [54].

### 3.6. Community Belonging during COVID-19

Table 7 presents the prevalence of perceived high community belonging and perceived stability/improvement in community belonging since the pandemic began. Overall, 66% of farmers reported high community belonging, which is significantly higher than Canadians in Spring 2021 (57.3%, *p* < 0.001), but not significantly different from Canadians in Fall 2020 (63.7%, *p* = 0.13) [54]. There were no significant gender differences within the farming sample, or between farming women and Canadian women in Spring 2021 or Fall 2020 [54]. However, 69.6% of farmers who identified as men reported high community belonging, which is significantly higher than Canadian men in Spring 2021 (58.2%, *p* < 0.001) and Fall 2020 (63.8%, *p* = 0.004) [54]. Seven in 10 (71.3%) farmers reported stable or improved community belonging since the pandemic began, and there were no significant gender differences.

### 3.7. Help Seeking during COVID-19

Approximately 1 in 5 (20.9%, *n* = 967) participants accessed any resources to help them manage their emotions, mental health, or use of alcohol or drugs during the COVID-19 pandemic. Notably, a significant gender difference was observed, as 32.3% of women sought help during the pandemic compared to 13.7% of men (*p* < 0.001).

## 4. Discussion

We conducted a national, online, cross-sectional survey of Canadian farmers (February–May 2021) using validated scales of generalized anxiety disorder (GAD-7), major depressive disorder (PHQ-9), perceived stress (PSS), burnout (MBI), alcohol use disorder (AUDIT), and resilience (CD-RISC), and perceived changes in these outcomes during the pandemic. Data were also collected on the impact of COVID-19 specific social and economic factors on mental health, help-seeking, and sense of community belonging through the pandemic.

### 4.1. Comparisons to 2016 Sample

The second National Survey of Farmer Mental Health in Canada builds upon the first national survey in 2015/16 [14,15], expands our knowledge through the addition of psychometric scales (e.g., coping, alcohol use), and adds important insights on the differential experiences of mental health by gender and the impacts of the COVID-19 pandemic on farmers’ mental health. The results from the 2015/16 national survey were generally agreed upon by farmers, agricultural organizations, industry, government, and mental health professionals as concerning and sparked considerable attention and discussion in Canadian agriculture. Regrettably, the 2021 survey data continue to document significant concerns with farmer mental health in Canada. Consistent with the 2016 survey results, farmers scored more severely on all mental health outcome measures than the general population. Farmers had higher mean scores for anxiety (GAD-7), depression (PHQ-9), perceived stress (PSS), and emotional exhaustion and cynicism MBI subscales, and lower resilience (CD-RISC) scale scores than scale norms. Because we used different scales in the 2021 survey than the 2016 survey to measure anxiety (GAD-7 vs. Hospital Anxiety and Depression Scale; HADS), depression (PHQ-9 vs. HADS), and resilience (CD-RISC 10-item vs. 25-item version), we cannot reliably compare results between the 2016 and 2021 samples. Notably, in 2016, farmers’ mean anxiety and depression subscale scores from the HADS were also significantly higher than scale norms, and farmers’ mean resilience (CD-RISC) scores on the CD-RISC 25-item version were also significantly lower than scale norms. Identical measures were used in both administrations of the survey for perceived stress (PSS) and burnout (MBI), and in both scales, no significant differences were observed between the overall 2021 and 2016 samples, other than the mean professional efficacy subscale score of the MBI was significantly lower in the 2021 sample than the 2016 sample (0.15 points difference). Therefore, although mental health awareness campaigns and mental health literacy programs within the agricultural sector have increased since the 2016 survey, we cannot report any improvement in population-level mental health outcomes in farmers since the first survey administration. However, it is possible that the mental health awareness campaigns have led farmers to become more self-aware of their mental health concerns to report them in this survey more accurately than in the 2016 survey. Regardless, greater efforts are needed to improve the mental health of Canadian farmers. National efforts must move beyond the awareness level to implement mental health (e.g., positive psychology training) and mental illness interventions that are developed for the agricultural context and are delivered by mental health professionals with experience or understanding in agriculture to meet farmers’ specific needs [18].

### 4.2. Comparisons to Canadians during COVID-19

Because national mental health polling was prioritized by Statistics Canada and Mental Health Research Canada throughout COVID-19, we could compare our results to nationally representative samples collected around the same time as our survey was active. Interestingly, more Canadians scored in the most severe categories, but more farmers scored in the moderate/harmful categories of anxiety (GAD-7), depression (PHQ-9), and alcohol use (AUDIT). A similar observation in the burnout data was made. Burnout is a syndrome characterized by high emotional exhaustion, high cynicism, and low professional efficacy. Interestingly, while farmers scored higher in emotional exhaustion and cynicism, they also scored higher in professional efficacy than the reference population. Hence, despite feelings of emotional exhaustion and the feeling of “indifference or a distant attitude towards work” that is associated with cynicism, participants nevertheless reported feelings of effectiveness at work and confidence in being “effective in getting things done” [41]. This observation of high emotional exhaustion and cynicism without low professional efficacy is likely indicative of participants being in a transitional state towards burnout (e.g., overextended profile) [63]. The findings of high/moderate classifications but not severe ones for anxiety (GAD-7, depression (PHQ-9), and alcohol use (AUDIT) may be explained by our participation requirements. Because we sampled those who currently identify as a farmer, our sample likely includes a higher proportion of people whose mental health or substance use concerns do not compromise their ability to remain in farming. Further in-depth qualitative research would be useful to investigate whether severe anxiety, depression, substance use, or burnout contribute to farmers leaving the profession–a notable decision for farmers who often view farming as a lifestyle rather than an occupation and are therefore less likely to consider changing occupations [64].

### 4.3. COVID-19 Impacts

Unsurprisingly, the pandemic was perceived to exacerbate issues with high stress, anxiety, depression, emotional exhaustion, cynicism, and alcohol use. Participants with high anxiety, depression, perceived stress, emotional exhaustion, and cynicism scores, and low resilience and professional efficacy scores, reported an increase in these symptoms since the beginning of the pandemic. This is consistent with national mental health polling, which reports worsening symptoms of anxiety and depression over the course of the pandemic [3,57], likely due to cumulative pandemic stressors [1] and in relation to severity of public health safety measures [65]. Further analyses are required to determine how the pandemic might have influenced the comorbidity of mental health outcomes in this sample. Brotto et al. (2021) found a relationship between increased alcohol consumption and symptoms of anxiety and depression during the pandemic, with the highest association between November 2020–March 2021 than any prior phase of the pandemic or prior to the pandemic [65]. Notably, many participants reported increased alcohol consumption since the pandemic began, which reflects a global trend of increased substance use during the pandemic, particularly during or following periods of high public health restrictions [66].

Because this is a cross-sectional study, we cannot determine whether the negative mental health outcomes we observed during the pandemic are more attributable to farming stressors and/or pandemic stressors, and/or their interactions. Although farmers perceived the pandemic to worsen mental health outcomes, farmers had significantly poorer self-rated mental health than Canadians in Spring 2021, and more farmers than Canadians reported stable or improved mental health since the pandemic began. We also know farmers scored more severely than the general population across mental health outcomes prior to the pandemic [15]. Additionally, community belonging may have been a protective factor for farmers’ mental health during the pandemic. Most farmers reported stable or increased community belonging since the pandemic began, and farmers, specifically farming men, reported higher community belonging than Canadians in Spring 2021. Strong community ties and high sense of belonging has been characteristic of rural communities in Canada prior to the pandemic [67], and community belonging is associated with strong mental health [67], feeling supported in times of change [68], and has been found to be a protective factor for Major Depressive Disorder during the pandemic [58]. Finally, our results suggest several occupational factors protected farmers’ mental health during the pandemic. For example, going outside was the COVID-19 related factor with the most positive impact on farmers’ mental health. The decline in mental health in the general population during the pandemic has been partly attributed to the decrease in opportunities for physical activity due to public health restrictions such as stay-at-home orders [69], and exposure to nature has been found to prevent the negative mental health implications of public health restrictions, including stay-at-home orders [70]. Fortunately, farmers often live on their farms and many farming activities are done outdoors, therefore, farmers may not have been as impacted by public health restrictions, and farmers’ mental health likely benefitted from being in nature during the pandemic. On the other hand, the COVID-19 related factor with the most negative impact on farmers’ mental health was daily COVID-19 news. Indeed, following the COVID-19 news closely has been associated with greater psychological distress [71]; however, during Spring 2021, social isolation was the leading COVID-19 stressor on Canadians’ mental health [3]. Therefore, although many farmers reported a negative impact of social isolation on their mental health, the socially isolated and outdoor nature of farming may have had a protective effect on farmers’ mental health during the pandemic [72].

### 4.4. Gender

Our findings raise important gender-based concerns and call for additional research and action to support the implementation of gender-based mental health programming in agriculture. In our sample, women scored more severely on measures of anxiety, depression, stress, and emotional exhaustion than men. This finding has been observed internationally and within Canada-women, regardless of sociodemographic characteristics (e.g., age, ethnicity) have consistently scored higher on measures of anxiety, depression, stress, loneliness throughout the pandemic [65]. Unsurprisingly, farming women also scored more severely on these measures compared to Canadian women during the pandemic. In addition to the occupational factors which might explain this finding, the poorer mental health of farming women compared to farming men may be due to women taking on disproportionate childcare duties during school closures, as the pandemic has led to more traditional gender roles in the agricultural industry [73].

Notably, no participants identified as nonbinary/gender-diverse in response to our open-ended gender question. Unfortunately, the gender distribution of farmers in Canada is unknown, because the Canadian Census of Agriculture collects data on sex instead of gender [74]. However, we know that mental health concerns are greater in gender-diverse and transgender individuals in the general population during the pandemic [65]. Future research which focuses on the mental health research of farmers who identify as gender-diverse, in addition to other equity-deserving groups like LGBTQIA+, would be helpful.

Over twice as many women reported seeking help for their mental health or substance use than men, highlighting an opportunity to help men decrease perceived barriers to help-seeking. Given our previous finding that farming women score more severely than men across all mental health outcomes, as previously recommended [75], it is important to not interpret higher help-seeking in women as an opportunity to reach farming men for mental health support. To reiterate, farming women are inundated with household and childcare responsibilities on top of their farm work, and women often work off the farm to subsidize farm bills [72]. To add the responsibility of organizing mental health support for men may present an additional burden on their own mental health. Previous qualitative work regarding mental health help-seeking of Canadian farmers [75] and farming men [76] has stressed the importance of mental health resource accessibility. Our findings echo their recommendations for gender-specific strategies to provide mental health supports to farmers.

The objective of this paper was to report prevalence estimates of mental health outcomes amongst farmers in Canada. Given the existing literature reporting gender as a risk factor for mental health concerns during the pandemic and the common gender differences in labour division in farmers in Canada, we also compared outcomes by gender. Comparisons of outcomes by other demographic variables are outside the scope of this article but will be investigated elsewhere.

### 4.5. Limitations

This is a cross-sectional, retrospective study with a convenience sample. Therefore, we have a snapshot of participants’ mental health and cannot infer causality. There is potential for recall bias because participants were asked to reflect on their mental health over the course of the pandemic. Although randomized sampling would have been ideal, we could not gain access to lists of farmers in Canada for research purposes. Our study sample was also limited to those who had access to internet, were members of an agricultural organization or were connected to agricultural social media. Hence, sampling bias may limit the generalizability of this sample to the total Canadian farming population. While this is the second national survey of farmer mental health in Canada, it is not a longitudinal study. Because responses were collected anonymously, we are unable to determine how many farmers participated in both the 2016 and 2021 surveys or to make intra-individual comparisons. We observed gendered differences in mental health outcomes, with women faring worse overall than men in our sample, however, it is possible that men underreported or did not experience the specific symptoms included in the scales despite having mental health concerns [77,78]. We strongly encourage future investigations of farmers’ mental health and gender. Finally, the survey was conducted strategically to be at a time of year with low farming stress (prior to planting season) to increase likelihood of participation. However, it is likely that farming stressors would increase over the planting and harvesting seasons for crop farmers, and during the extreme hot and cold seasons for livestock farmers; hence, our estimates may be underestimated for the yearly average.

### 4.6. Implications

We emphasize the importance of implementing national mental health interventions for Canadian farmers beyond awareness campaigns. Accessible and affordable mental health services delivered by mental health professionals with experience in agriculture, such as the Farmer Wellness Initiative in Ontario [79], address many of the barriers to help-seeking farmers in Canada have previously identified [75]. Mental illness interventions, such as the Farmer Wellness Initiative in Ontario, and mental health promotion programs, like the Ripple Effect intervention for suicide stigma reduction [80], and the Campfire platform delivered by the National Centre for Farmer Health in Australia [81] are needed at the national scale.

Given the low scores for resilience observed in this study, implementing positive psychology interventions in farming populations as a companion to tailored mental health services may be useful for universal mental health promotion. There is evidence that online positive psychology interventions have been effective during the pandemic to strengthen the resilience during stay-at-home orders or social distance requirements [82,83] and with frontline workers [84]. Additionally, positive psychology interventions are recommended for use in the public health context as the first step in a stepped care approach and are also effective when delivered in a self-directed, online format [85,86] and through phone apps [87]. Given the recent prioritization of high speed internet access for people living in rural and remote areas of Canada [88], online delivery of interventions may become preferable for farmers in the future due to their convenience and anonymity [75].

Although this survey was conducted in the context of COVID-19 and pandemic stressors were measured, we caution against solely attributing our findings to the pandemic, as it is likely that the pandemic exacerbated existing mental health concerns related to farming stressors. Indeed, we did not observe any improvement in mental health outcomes between 2016 and 2021 despite knowledge of mental health awareness and literacy efforts within the agricultural sector. Finally, further research investigating how farming affects mental health differently by gender will be helpful to improve existing farmer mental health resources and develop necessary gender-specific resources.

## 5. Conclusions

Overall, the findings from the second National Survey of Farmer Mental Health reflect significant concerns with the mental health and well-being of farmers in Canada. Farmers scored higher on measures of anxiety and depression than the Canadian general population during COVID-19, and scored higher on measures of perceived stress, emotional exhaustion and cynicism (two components of burnout) and alcohol use, and lower on resilience, than population norms prior to COVID-19. Participants perceived the COVID-19 pandemic as detrimental to their mental health, as participants identified specific COVID-19 variables which negatively impacted their mental health, and reported experiencing symptoms of anxiety, depression, perceived stress, emotional exhaustion, and cynicism more often since the pandemic began. Notably, participants who identified as women scored more severely on measures of anxiety, depression, perceived stress, and emotional exhaustion than farming men and Canadian women. While great strides have been made in terms of awareness and programming since the first cycle of this survey was conducted in 2016, the data suggest that we must further expand our efforts to include accessible, gender-specific mental health resources tailored for farmers and available nationally. Additionally, future research is encouraged to develop positive psychology interventions for protection against daily farming stressors and prevent negative mental health impacts of future pandemics or farming catastrophes.

## Figures and Tables

**Table 1:** Complete list of individual items measuring COVID-19 specific factors, from Mental Health Research Canada’s Mental Health During COVID-19 Outbreak polls [3,52].

During the Current Coronavirus (COVID-19) Outbreak in Canada, Please Rate Each of the Following in Terms of the Impact They Are Currently Having on Your Mental Health, If Any:
The possibility of you catching COVID-19
The possibility of a family member catching COVID-19
Social isolation/being apart from others
The difficulties and challenges of getting necessities (groceries, prescriptions, other household items)
Communicating with family/friends outside of your household (via phone, email, video chats)
Interacting with members of your household in-person
The economic downturn
The possibility of not being able to pay household/farm bills in full owed in 2020
Daily news about the coronavirus (COVID-19) pandemic
Physical activity/exercise
Social media, such as Facebook, Twitter, Instagram, etc.
Entertainment, such as television, movies, music, podcasts, etc.
Reading books, short stories, essays, etc. that are not about the COVID-19 pandemic
Going outside/walking/hiking
Pets

**Table 2:** Demographics of study participants (February–May 2021) (*n* = 1167), and Canadian farmers from the 2021 Census of Agriculture (*n* = 262,642) [56].

Characteristic	Participants	Farmers in Canada 2020 [56]	
Age (years)	*n* = 968	*n* = 262,470	
<35	177 (18.3)	22,640 (8.6)	
35–54	408 (42.1)	81,040 (30.9)	
55+	383 (39.6)	158,790 (60.5)	
Gender	*n* = 984	Sex ^a^	*n* = 262,450 ^a^
Women	366 (39.3)	Female ^a^	79,795 (30.4) ^a^
Men	565 (60.7)	Male ^a^	182,655 (69.6) ^a^
Province	*n* = 1084	*n* = 262,470	
British Columbia	19 (1.8)	23,685 (9.0)	
Alberta	71 (6.5)	57,195 (21.8)	
Saskatchewan	59 (5.4)	44,140 (16.8)	
Manitoba	34 (3.2)	19,470 (7.4)	
Ontario	537 (49.5)	67,400 (25.7)	
Quebec	323 (29.8)	42,280 (16.1)	
Newfoundland and Labrador	3 (0.3)	440 (0.2)	
New Brunswick	18 (1.7)	2475 (0.9)	
Nova Scotia	12 (1.1)	3785 (1.4)	
Prince Edward Island	4 (0.4)	1600 (0.6)	
Yukon	3 (0.3)	-	
Northwest Territories and Nunavut	1 (0.1)	-	
Work off farm	*n* = 1162	*n* = 262,642	
Yes	416 (35.8)	125,280 (47.7)	
No	746 (64.2)	137,362 (52.3)	
If yes, need to work off farm to pay monthly expenses	*n* = 410	-	
Yes	314 (76.6)	-	
No	96 (23.4)	-	
Commodity ^b^	*n* = 1150	*n* = 189,874	
Beef, including feedlots	331 (28.8)	39,633 (20.9)	
Dairy and milk	404 (35.1)	9403 (5.0)	
Hog and pig	151 (13.1)	3016 (1.6)	
Poultry and egg	258 (22.4)	5296 (2.8)	
Sheep and goat	119 (10.3)	3575 (1.9)	
Other animal production	299 (26.0)	15,873 (8.4)	
Oilseed and grain	612 (53.2)	65,135 (34.3)	
Vegetable and melon	119 (10.3)	5076 (2.7)	
Fruit and tree nut	89 (7.7)	7101 (3.7)	
Greenhouse, nursery, and floriculture	77 (6.7)	5256 (2.8)	
Other crop	495 (43.0)	30,510 (16.1)	
Language	*n* = 1167	-	
English	824 (70.6)	-	
French	343 (29.4)	-	
Relationship Status	*n* = 950	-	
Single	74 (7.5)	-	
Committed relationship–not living together	28 (2.9)	-	
Cohabitating/living together	28 (2.9)	-	
Married/common law	804 (82.0)	-	
Separated/divorced	23 (2.3)	-	
Widowed	14 (1.4)	-	
Married & widowed	8 (1.0)	-	
Previous mental illness	*n* = 975	-	
Yes	150 (15.4)	-	
No	825 (84.6)	-	
If yes, currently being treated (medication, counselling) for mental illness	*n* = 150	-	
Yes	89 (59.3)	-	
No	61 (40.7)	-	

^a^ Sex is reported as the 2021 Census of Agriculture collected data on sex, not gender. ^b^ Sample population reported all commodities (hence, totals add to >100%) whereas Statistics Canada reports only primary commodity.

**Table 3:** Descriptive statistics (*n*, mean, standard deviations) for each validated scale used for the overall 2021 sample and by gender, and with comparisons to scale population norms and 2015/2016 survey data, where available.

	Farmers 2021			Scale Norm			Farmers 2016		
	*n*	Mean (SD)	*p*-Value ^a^	*n*	Mean (SD)	*p*-Value ^b^	*n*	Mean (SD)	*p*-Value ^c^
GAD-7									
Total	980	6.12 (4.9)	-	5030	2.97 (3.4)	<0.001	-	-	-
Men	563	5.45 (4.6)	<0.001	2332	2.66 (3.2)	<0.001	-	-	-
Women	364	7.16 (5.2)		2698	3.20 (3.5)	<0.001	-	-	-
PHQ-9									
Total	978	5.89 (5.3)	-	5018	2.91 (3.5)	<0.001	-	-	-
Men	562	5.25 (4.9)	<0.001	2326	2.70 (3.5)	<0.001	-	-	-
Women	363	6.87 (5.6)		2692	3.10 (3.5)	<0.001	-	-	-
PSS									
Total	1110	18.72 (7.03)		2270	13.0 (6.34)	<0.001	1127	18.9 (4.9)	0.48
Men	560	17.46 (7.15)	<0.001	926	12.1 (5.90)	<0.001	677	18.3 (4.9)	0.02
Women	363	20.46 (6.98)		1344	13.7 (6.60)	<0.001	295	20.1 (4.9)	0.44
MBI									
Total	973	18.72 (7.0)	-	2270	13.0 (6.3)	<0.001	1127	18.9 (4.9)	0.50
Men	560	17.46 (7.1)	<0.001	926	12.1 (5.9)	<0.001	677	18.3 (4.9)	0.02
Women	360	20.46 (7.0)		1344	13.7 (6.6)	<0.001	295	20.1 (4.9)	0.44
Emotional Exhaustion									
Total	973	2.64 (1.6)	-	47,800	2.26 (1.5)	<0.001	1075	2.68 (1.6)	0.57
Men	560	2.46 (1.6)	<0.001	-	-	-	670	2.57 (1.6)	0.23
Women	360	2.92 (1.6)		-	-	-	290	2.91 (1.6)	0.94
Cynicism									
Total	973	2.20 (1.4)	-	47,752	1.74 (1.4)	<0.001	1005	2.12 (1.5)	0.22
Men	560	2.16 (1.4)	0.36	-	-	-	647	2.06 (1.4)	0.22
Women	360	2.26 (1.5)		-	-	-	287	2.27 (1.5)	0.93
Professional Efficacy									
Total	973	4.70 (1.1)	-	47,843	4.34 (1.2)	<0.001	1008	4.85 (1.0)	0.001
Men	560	4.80 (1.1)	0.13	-	-	-	651	4.88 (1.0)	0.19
Women	360	4.69 (1.1)		-	-	-	288	4.75 (1.1)	0.49
AUDIT									
Total	948	4.46 (4.0)	-	3623	3.61 (3.1)	<0.001	-	-	-
Men	546	4.86 (4.4)	<0.001	1685	4.47 (3.6)	0.06	-	-	-
Women	354	3.73 (3.4)		1938	2.71 (2.1)	<0.001	-	-	-
CD-RISC 10									
Total	980	24.66 (6.2)	-	764	31.80 (5.4)	<0.001	-	-	-
Men	562	25.04 (6.1)	0.02	218	33.53 (4.6)	<0.001	-	-	-
Women	365	24.10 (6.2)		546	31.08 (5.6)	<0.001	-	-	-

^a^ Statistical comparison within 2021 data. ^b^ Statistical comparison of 2021 data with population normative data. ^c^ Statistical comparison of 2021 data with 2015/2016 survey data.

**Table 4:** Prevalence of positive GAD screen (GAD-7 score >10) for the overall 2021 sample and by gender, and in comparison to Canadian general population from Statistics Canada’s Survey of COVID-19 and Mental Health (SCMH) cycles 1 (Spring 2020) and 2 (Fall 2021) [57].

	Farmers 2021			SCMH 2021		SCMH 2020	
Positive GAD Screen (GAD-7 Score > 10)	*n*	%	*p*-Value ^a^	%	*p*-Value ^b^	%	*p*-Value ^c^
Total	238	26.8	-	15.0	<0.001	13.0	<0.001
Men	101	21.0	<0.001	-	-	12.0	<0.001
Women	114	34.9		-	-	17.0	<0.001

^a^ Statistical comparison within 2021 data. ^b^ Statistical comparison of 2021 data with Canadian general population in Spring 2021. ^c^ Statistical comparison of 2021 data with Canadian general population in Fall 2020.

**Table 5:** Prevalence of positive MDD screen (PHQ-9 score >10) for the overall 2021 sample and by gender, and in comparison to Canadian general population from Statistics Canada’s Survey of COVID-19 and Mental Health (SCMH) cycles 1 (Spring 2020) and 2 (Fall 2021) and prior to the pandemic from Canadian Community Health Survey (CCHS) data (2015–2019) [57].

	Farmers 2021			SCMH 2021		SCMH 2020		CCHS 2015–2019	
Positive MDD Screen (PHQ-9 Score > 10)	*n*	%	*p*-Value ^a^	%	*p*-Value ^b^	%	*p*-Value ^c^	%	*p*-Value ^d^
Total	209	23.6	-	19.0	<0.001	15.2	<0.001	6.7	<0.001
Men	98	20.5	<0.001	-	-	12.6	<0.001	5.8	<0.001
Women	94	28.1		-	-	17.5	<0.001	7.5	<0.001

^a^ Statistical comparison within 2021 data. ^b^ Statistical comparison of 2021 data with Canadian general population in Spring 2021. ^c^ Statistical comparison of 2021 data with Canadian general population in Fall 2020. ^d^ Statistical comparison of 2021 data with Canadian general population between 2015 and 2019.

**Table 6:** Prevalence of high self-rated mental health and perceived stability/improvement in mental health since the pandemic began for the overall 2021 sample and by gender in comparison to the Canadian general population from Statistics Canada’s Survey of COVID-19 and Mental Health (SCMH) cycles 1 (Spring 2020) and 2 (Fall 2021).

	Farmers 2021			SCMH 2021		SCMH 2020	
	*n*	%	*p*-Value ^a^	%	*p*-Value ^b^	%	*p*-Value ^c^
High self-rated mental health							
Total	452	46.3	-	51.5	0.02	59.9	<0.001
Men	297	52.8	<0.001	54.0	0.69	64.5	<0.001
Women	133	36.4		49.3	0.002	55.7	<0.001
Perceived stability/improvement in mental health							
Total	645	66.0	-	58.1	<0.001	66.5	0.72
Men	391	69.4	0.005	61.1	<0.001	71.0	0.42
Women	220	60.3		55.3	0.06	62.3	0.42

^a^ Statistical comparison within 2021 data. ^b^ Statistical comparison of 2021 data with Canadian general population in Spring 2021 ^c^ Statistical comparison of 2021 data with Canadian general population in Fall 2020.

**Table 7:** Prevalence of high self-rated community belonging and perceived stability/improvement in community belonging since the pandemic began for the overall 2021 sample and by gender in comparison to the Canadian general population from Statistics Canada’s Survey of COVID-19 and Mental Health (SCMH) cycles 1 (Spring 2020) and 2 (Fall 2021).

	Farmers 2021			SCMH 2021		SCMH 2020	
	*n*	%	*p*-Value ^a^	%	*p*-Value ^b^	%	*p*-Value ^c^
High self-rated community belonging							
Total	643	66.0	-	57.3	<0.001	63.7	0.24
Men	389	69.6	0.68	58.2	<0.001	63.8	0.02
Women	220	60.3		56.7	0.26	63.6	0.33
Perceived stability/improvement in community belonging							
Total	696	71.3	-	-	-	-	-
Men	408	72.9	0.03	-	-	-	-
Women	246	67.4		-	-	-	-

^a^ Statistical comparison within 2021 data ^b^ Statistical comparison of 2021 data with Canadian general population in Spring 2021 ^c^ Statistical comparison of 2021 data with Canadian general population in Fall 2020.

## Data Availability

Data sharing is not applicable to this article because the research participants did not consent to data sharing.
